# Disrupting enzyme fluidity

**DOI:** 10.7554/eLife.65221

**Published:** 2021-01-25

**Authors:** Ganesh Srinivasan Anand

**Affiliations:** Department of Chemistry and Huck Institute of Life Sciences, Pennsylvania State UniversityUniversity ParkUnited States

**Keywords:** bruton tyrosine kinase, kinase inhibitor, drug resistance, allostery, hydrogen/deuterium exchange mass spectrometry, nuclear magnetic resonance, None

## Abstract

A combination of X-ray crystallography, NMR, and mass spectrometry has revealed how diverse small-molecule inhibitors bind Bruton’s tyrosine kinase and alter the conformation of this enzyme.

**Related research article** Joseph RE, Amatya N, Fulton DB, Engen JR, Wales TE, Andreotti A. 2020. Differential impact of BTK active site inhibitors on the conformational state of full-length BTK. *eLife* **9**:e60470. doi: 10.7554/eLife.60470

The switching of enzymes between active and inactive states, a process known as enzyme regulation, is crucial in cell biology, and the breakdown of the process has been implicated in many diseases. A number of small-molecule inhibitors work by blocking enzyme function, but efforts to evaluate the efficacy of such inhibitors have been hampered by the lack of a detailed understanding of how they work. For example, some small-molecule inhibitors work by making localized changes to the shape of the enzyme at the site where they bind, whereas others work by inducing changes in another part of the enzyme, a phenomenon known as allostery.

A clearer picture of allostery requires detailed knowledge of enzyme function and the underlying protein dynamics (Henzler-Wildman and Kern, 2007). Now, in eLife, Amy Andreotti (Iowa State University), Thomas Wales (Northeastern University) and colleagues – Raji Joseph, Neha Amatya, Bruce Fulton and John Engen – report on the effects of five different small-molecule inhibitors on an enzyme called Bruton’s tyrosine kinase (BTK; Joseph et al., 2020).

BTK is a kinase that regulates the immune responses of B- and T-cells, and blocking its activity can help suppress inflammatory responses and treat lymphomas and leukemias (Kim, 2019). High-resolution snapshots of the active and inactive states of BTK have been previously obtained using X-ray crystallography (Marcotte et al., 2010; Kuglstatter et al., 2011; Xing and Huang, 2014). Similar to other kinases, an important feature in BTK is a switch called a ‘Glu-Lys switch’ (Taylor et al., 1993). When the critical glutamate (Glu) in the switch is positioned close to a specific lysine (Lys) in the catalytic site, the enzyme is more active. When the enzyme is inactive, it adopts a different shape where the same glutamate is further from the lysine.

While X-ray crystallography can provide information on the active and inactive conformations of enzymes at high resolution, additional techniques are needed to understand how enzymes and small-molecule inhibitors interact in solution, so Joseph et al. combined X-ray crystallography with nuclear magnetic resonance (NMR) and amide hydrogen/deuterium exchange mass spectrometry (HDXMS). NMR provides a global overview of conformation, including the transitions between the active and inactive state, while HDXMS localizes conformational changes at a peptide level.

The experiments revealed that BTK exists in an ensemble of conformations, encompassing the inactive and active states of the enzyme (Figure 1). Joseph et al. then explored how five different small-molecule inhibitors (ibrutinib, dasatinib, GDC-0853, CGI1746 and CC-292) interacted with BTK, and found that each inhibitor resulted in varying ratios of inactive and active conformations. These results establish that BTK changes the likelihood of being in a specific conformation within its ensemble, rather than operating as a discrete on/off switch, which is consistent with the idea that proteins exist in several conformations of varying activity (Onuchic and Wolynes, 2004). This work also supports the view that small-molecule inhibitors may favor certain conformations in an ensemble over others (Boehr et al., 2009; Kar et al., 2010). Joseph et al. also examined a mutation in BTK that confers B-cells with resistance to one of the inhibitors: the cancer drug ibrutinib. They found that this mutation disrupts the inactive conformation of BTK, making the enzyme more active and leading to more aggressive lymphomas that can evade the drug.

**Figure 1.**
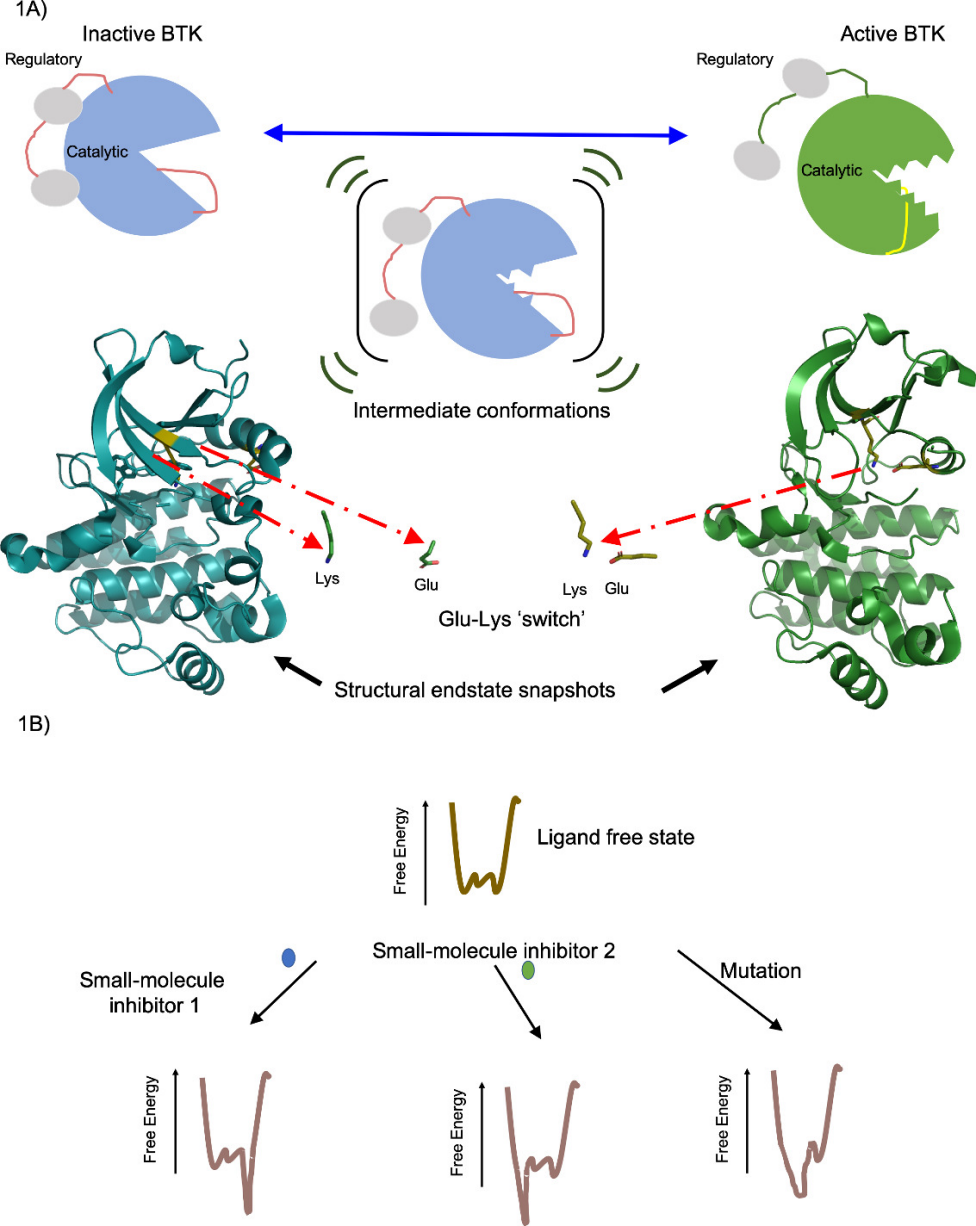
Active and inactive forms of the enzyme BTK. (**A**) Top: In solution BTK populates an ensemble of inactive (left), intermediate (middle) and active (right) conformations. These schematics show the regulatory domain (red), the catalytic domain (blue or green) and activation loop (red or yellow). Activation involves the regulatory domain moving away from the catalytic domain, and the activation loop moving away from the Glu-Lys switch (not shown) in the catalytic domain. Bottom: Structure of the catalytic domain in the inactive (blue, left) and active (green, right) conformations showing the Glu-Lys switch turned away in the inactive state and positionally close together in the active states (Marcotte et al., 2010). These structures of inactive (PDB ID: 3GEN) and active (PDB ID: 3K54) BTK were rendered using the PyMOL Molecular Graphics System, Version 2.0 Schrödinger, LLC. (**B**) The effect of a small-molecule inhibitor on an enzyme can be visualized in terms of the way it changes the free energy of an ensemble of interchangeable conformational states of the enzyme. In the absence of the small-molecule inhibitors, the free energy (vertical axis) is different for each of the individual conformations (horizontal axis); the lower the free energy, the more likely the enzyme is likely to exist in that conformation. In this example, small-molecule inhibitor 1 (blue) lowers the free energy to the right of the graph, favoring these specific conformations, whereas small-molecule inhibitor 2 (green) lowers the free energy to the left of the graph, favoring those specific conformations. Mutations in the enzyme can also change the free energy to favor certain conformations. Knowing how different small molecules influence the free energy of the enzyme could help develop new small-molecule inhibitors.

This work underscores the limitations of using structural snapshots from X-ray crystallography or cryo-electron microscopy alone to map small-molecule interaction sites, or to describe allosteric effects. It also highlights how combining NMR and HDXMS with static structural data will lead to more complete descriptions of drug-enzyme interactions. More generally, combining structural, dynamic and computational approaches will help researchers to design inhibitor drugs that are not rendered ineffective by disease-resistant mutations.
